# Overexpression of cathepsin K during silica-induced lung fibrosis and control by TGF-β

**DOI:** 10.1186/1465-9921-6-84

**Published:** 2005-07-27

**Authors:** Sybille van den Brûle, Pierre Misson, Frank Bühling, Dominique Lison, François Huaux

**Affiliations:** 1 Unit of Industrial Toxicology and Occupational Medicine, Université catholique de Louvain, Clos Chapelle-aux-Champs, 30.54, 1200 Brussels, Brussels, Belgium; 2 Institute of Immunology, Otto-von-Guericke-University Magdeburg, Leipziger-Str. 44, 39120 Magdeburg, Germany

## Abstract

**Background:**

Lung fibrosis is characterized by tissue remodeling resulting from an imbalance between synthesis and degradation of extracellular organic matrices. To examine whether cathepsin(s) (Cat) are important in the development of pulmonary fibrosis, we assessed the expression of four Cat known for their collagenolytic activity in a model of silica-induced lung fibrosis.

**Methods:**

Different strains of mice were transorally instilled with 2.5 mg crystalline silica or other particles. Cat expression (Cat K, S, L and B) was quantified in lung tissue and isolated pulmonary cells by quantitative RT-PCR. *In vitro*, we assessed the effect of different cytokines, involved in lung inflammatory and fibrotic responses, on the expression of Cat K by alveolar macrophages and fibroblasts.

**Results:**

In lung tissue, Cat K transcript was the most strongly upregulated in response to silica, and this upregulation was intimately related to the fibrotic process. In mouse strains known for their differential response to silica, we showed that the level of Cat K expression following silica treatment was inversely related to the level of TGF-β expression and the susceptibility of these strains to develop fibrosis. Pulmonary macrophages and fibroblasts were identified as Cat K overproducing cells in the lung of silicotic mice. *In vitro*, Cat K was downregulated in mouse and human lung fibroblasts by the profibrotic growth factor TGF-β1.

**Conclusion:**

Altogether, these data suggest that while Cat K may contribute to control lung fibrosis, TGF-β appears to limit its overexpression in response to silica particles.

## Background

Tissue remodeling is a dynamic process common to several pulmonary disorders, such as asthma and lung fibrosis. It generally follows an inflammatory injury and involves an unbalanced repair process characterized by an inappropriate production/degradation of the organic matrix, which leads to abnormal lung architecture and impairment of lung function [1]. Remodeling involves destruction of basement membranes as well as of elastic fibers, and the exaggerated accumulation of organic extracellular matrices (ECM) [1-5]. During the fibrogenic process, the pre-existing equilibrium between matrix synthesis and degradation in the healthy lung [6] is disrupted, leading to an excessive accumulation of ECM. The secretion of growth factors, such as transforming growth factor-β (TGF-β) and platelet-derived growth factor (PDGF), together with the modified expression of matrix degrading-related enzymes contribute to the increased production by fibroblasts and pulmonary accumulation of ECM, such as collagen [1].

Matrix metalloproteases (MMPs) have been extensively studied for their role in ECM turnover in the lung and other organs [7,8]. Several MMPs were found to be abnormally regulated in human fibrotic diseases [9,10] and rodent models of fibrosis [11-13]. Although the expression of most MMPs was observed to be increased in fibrotic lungs, the expression of collagenases (MMP-1, 8, 13) appears to depend on the model or type of pathogenesis studied and the stage of the disease. The simultaneously increased expression of tissue inhibitors of metalloproteases (TIMPs) led several authors to suggest that an imbalance between MMPs and TIMPs occurring during fibrogenesis could lead to abnormal lung remodeling [11,12,14,15]. Despite clues pointing to MMPs/TIMPs as important players in the control of fibrosis, none of them has been shown so far to exert a protective function in this process *in vivo *[16,17].

Since they have also been involved in the turnover/degradation of ECM [18], lysosomal cysteine proteases could also apply to play a role in the development of lung fibrosis. One of them, cathepsin K (Cat K), is the most potent mammalian collagenase compared to other cysteine proteases (Cat B, L and S) and MMPs [19,20]. Cat K plays a pivotal role in bone remodeling. Indeed, mutations in the Cat K gene were found to be responsible of pycnodysostosis in humans [21] and of a similar bone phenotype in mouse [22]. In a murine model of lung fibrosis induced by bleomycin, this cathepsin was found to be induced in the lung [23]. Recently, it was suggested to exert a protective role against matrix deposition during pulmonary fibrosis, since lungs of Cat K deficient mice accumulated more collagen than wild type animals in response to bleomycin [24].

The purpose of this work was to identify lysosomal cysteine proteases potentially important in the development of pulmonary fibrosis in a murine model induced by the instillation of crystalline silica particles. Our study revealed that Cat K transcripts are highly increased in the lungs after silica treatment compared to Cat S, L and B and that this upregulation is specific to the fibrotic process. We also compared Cat K expression in "fibrosis-prone" and "fibrosis-resistant" mouse strains, and identified cells responsible for Cat K upregulation in the silicotic lung. Finally, the regulation of Cat K expression by growth factors involved in the inflammatory and/or fibrotic reactions was studied *in vitro *in both mouse and human fibroblasts.

## Methods

### Animals and instillation method

C57BL/6 and BALB/c female mice were obtained from the local breeding facility of the Ludwig Institute (Brussels, Belgium). NMRI female mice were purchased from Charles River Laboratories (Brussels, Belgium). Animals were housed in positive pressure air-conditioned units (25°C, 50% relative humidity) on a 12 h light/dark cycle. Eight to ten week-old mice were used. Crystalline silica (DQ12, d_50 _= 2.2 μm, a gift from Dr. Armbruster, Essen, Germany), manganese dioxide (MnO_2_) or tungsten carbide (WC) particles were heated at 200°C for 2 h before use to remove any trace of endotoxin. For instillation, animals were anesthetized with a mix of Ketalar (n.v. Warner-Lambert, Zaventem, Belgium) and Rompun (Bayer, Leverkusen, Germany) (respectively 1 and 0.2 mg/mouse i.p.). Particles were suspended in sterile phosphate buffered saline (PBS) and 2.5 mg particles/mouse (60 μl/mouse) were instilled into the lungs via the trachea by transoral instillation. Control mice were instilled with a corresponding volume of PBS. At selected time intervals, mice were sacrificed with an overdose of sodium pentobarbital (11 mg/animal given i.p.).

### Lung homogenates

Whole lungs were perfused with 5 ml sterile 0.9 % NaCl and then excised. The left lobe was placed in Trizol (Invitrogen, Paisley, USA) for subsequent RNA extraction and the right lobes transferred to 3 ml cold PBS. For the Cat K activity test, entire lungs were collected in PBS. Lungs in PBS were homogenized on ice with an Ultra-Turrax T25 homogenizer (Janke & Kunkel, Brussels, Belgium) and stored at -80°C.

### Bronchoalveolar lavage (BAL) cells and macrophage enrichment

Bronchoalveolar lavages were performed by cannulating the trachea and infusing the lungs with four volumes of 1 ml sterile 0.9 % NaCl. Lavages collected from control or treated mice were pooled and centrifuged 10 min at 400 *g *(4°C). Cell pellets were rinsed with sterile PBS. To determine the proportion of macrophages, cells were pelleted onto glass slides by cytocentrifugation and counted by light microscopy after Diff-Quick staining (200 cells counted, Dade Behring AG, Düdingen, Switzerland). For RNA extraction of total BAL cells, RLT lysis buffer (RNeasy mini kit, Qiagen, Maryland, USA) was directly added to the cell pellets. For macrophage enrichment, cell pellets were resuspended in an adequate volume of Dulbecco's modified Eagle's medium (DMEM, Invitrogen) supplemented with 10 % fetal bovine serum (FBS, Invitrogen), 2 mM L-glutamine (Invitrogen), 50 U/ml penicillin and 50 μg/ml streptomycin (Invitrogen) to obtain a suspension of 10^6 ^macrophages/ml. Four ml of this alveolar cell suspension were seeded into 6-well culture plates and incubated at 37°C under 5% CO_2_. After 2 h, the cultures were washed twice with PBS to remove non-adherent cells, and adherent cells were lysed with RLT buffer.

### Fibroblast culture

Perfused whole lungs were minced with scissors and suspended in DMEM containing 10 % FBS, 50 U/ml penicillin and 50 μg/ml streptomycin (10 ml medium/lung). Twenty ml of this suspension was transferred to a flat tissue culture flask and incubated at 37°C under 5% CO_2_. The medium was replaced every week. After 2 to 3 weeks, cells were washed twice with 10 ml PBS, detached with 0.05 % trypsin (10 ml, Invitrogen) and then collected with 10 ml DMEM supplemented with 10 % FBS. The cell suspension was passed trough a sterile 70 μm nylon filter and centrifuged 10 min at 260 *g *(4°C). After resuspension of cell pellets in DMEM, cell number and viability were determined with trypan blue (Sigma, St Louis, USA). Suspensions were adjusted to 5.10^5 ^fibroblasts/3 ml of DMEM containing 10 % FBS, 50 U/ml penicillin and 50 μg/ml streptomycin. Aliquots of 3 ml were seeded into 6-well culture plates and incubated at 37°C under 5% CO_2_. When no treatment was applied to the fibroblasts, the cells were washed after 24 h and lysed with RLT buffer. To test the effect of cytokines on Cat K expression, cells were grown to pre-confluence, rinsed twice with PBS and then supplemented with fresh medium (DMEM containing 2 mM L- glutamine, 200 μM proline (Sigma), 50 μg/ml L-ascorbic acid (Sigma), 50 U/ml penicillin and 50 μg/ml streptomycin) alone (non-treated) or containing recombinant human interleukin-1β (IL-1β, Roche, Vilvoorde, Belgium), mouse tumor necrosis factor-α (TNF-α, R&D Systems), recombinant mouse IL-4 (R&D Systems, Minneapolis, USA), recombinant mouse IL-9 [25], prostaglandin E2 (PGE2, Sigma) or human TGF-β1 (R&D Systems). After 24 h incubation, fibroblasts were washed with PBS and lysed with RLT buffer. Human fibroblasts from healthy lung tissue were obtained as described in Bühling et al. [24] and incubated with TGF-β1. After 48 h, fibroblasts were washed with PBS and lysed with RLT buffer for subsequent RNA extraction.

### Hydroxyproline assay

Collagen deposition was estimated by measuring hydroxyproline content in lungs homogenized in PBS. Hydroxyproline was assessed by high-pressure liquid chromatography analysis on hydrolyzed lung homogenates (6 N HCl at 108°C during 24 h) as previously described [26].

### Total TGF-β1 lung content

Total TGF-β1 lung contents were measured in lung homogenates by ELISA (Enzyme-linked immunosorbent assay) using the Quantikine human TGF-β1 immunoassay (R&D systems, Wiesbaden-Nordenstadt, Germany) according to manufacturer's instructions.

### Total RNA extraction and quantification of cathepsin transcripts

Perfused left lung lobes were homogenized on ice in 3 ml Trizol using an Ultra-Turrax T25. Total RNA extraction was performed according to Trizol manufacturer's instructions. RNA from centrifuged BAL cells and cell cultures was extracted with the RNeasy mini kit (Qiagen). Residual DNA contamination was removed by treatment with DNA-free (Ambion, Austin, USA). Between 100 ng and 1 μg of RNA was reverse transcribed with Superscript RNase H^- ^Reverse Transcriptase (Invitrogen) with 350 pmol random hexamers (Eurogentec, Seraing, Belgium) in a final volume of 25 μl. Resulting cDNA was then diluted 50× and used as template in subsequent polymerase chain reaction (PCR). Sequences of interest were amplified using the following forward primers: AGA GGG AAA TCG TGC GTG AC (mouse β-actin), ACT TGG GAG ACA TGA CCA GTG A (mouse Cat K), CAC TGA GGT GAA ATA CCA GGG TTC (mouse Cat S), CTC TGG AGC ATG GAG CTT CTG (mouse Cat B), CTG TGA AGA ACC AGG GCC AG (mouse Cat L), and reverse primers: CAA TAG TGA TGA CCT GGC CGT (mouse β-actin), TCT TGA CTG GAG TAA CGT ATC CTT TC (mouse Cat K), GAT GTA CTG GAA AGC TTC GGT CA (mouse Cat S), CGC TGT AGG AAG TGT ACC CAA AG (mouse Cat B), CCT TGA GCG TGA GAA CAG TCC (mouse Cat L). PCR was primarily performed with Platinum Taq DNA polymerase (Invitrogen) according to manufacturer's instructions with the following temperature program: 2 min 94°C, (30 s 94°C, 30 s 55°C, 20 s 72°C) ×40, 5 min 72°C. Amplified DNA fragments were purified from a 1.5 % agarose gel with Nucleospin Extract (Macherey-Nagel, Düren, Germany) and then serially diluted to serve as standards in real-time PCR. Reverse transcribed mRNAs were finally quantified by real-time PCR using SYBR Green technology on an ABI Prism 7000 Sequence Detection System (Applied Biosystems, Foster City, USA) according to the following program: 2 min 50°C, 10 min 95°C, (15 s 95°C, 1 min 60°C) ×40. Five μl of diluted cDNA or standards were amplified with 300 nM of the described primers using SYBR Green PCR Master Mix (Applied Biosystems) in a total volume of 25 μl. PCR product specificity was verified by taking a dissociation curve and by agarose gel electrophoresis. RT-PCR on RNA isolated from human fibroblasts was performed as previously described [24]. Results were calculated as a ratio of cathepsin expression to the expression of the reference gene, β-actin.

### Cat K enzymatic activity

Whole lung homogenates were sonicated on ice for 3 s and then centrifuged 5 min at 2600 *g *(4°C). Assays were performed on resulting supernatants as previously described [27,28]. Briefly, 200 μl samples were incubated 15 min with Cat K substrate, Z-GPR-AMC (80 μM, Biomol, Plymouth Meeting, USA) in presence of the cysteine proteases inhibitor, E64 (16 μM, Biomol) or the Cat B specific inhibitor, CA-074 (16 μM, Biomol) in a total volume of 1 ml. The reaction was terminated by the addition of 2 ml stop buffer and the resulting fluorescence was measured using a SPF-500 ratio spectrofluorometer (Aminco, Silver Spring, USA, excitation 365 nm, emission 440 nm). Cat K enzymatic activities are presented as the difference of fluorescence intensities between measurements in presence of CA-074 and in presence of E-64.

### Statistics

Differences were evaluated using t tests and one-way analysis variance, followed by Dunnett's test, as appropriate. Statistical significance was considered at *P *< 0.05. Data analysis was performed with GraphPad InStat version 3.05 for Windows 95/NT (GraphPad Software, San Diego, USA).

## Results

### Cat K is more strongly upregulated than Cat S, L and B during silica-induced fibrosis

**Figure 1:**
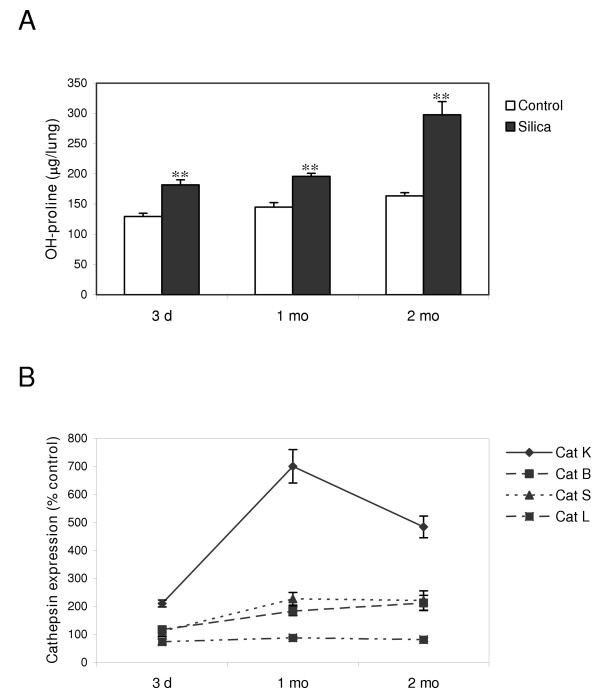
Cat K mRNA is strongly upregulated in the lungs of silica-treated mice. C57BL/6 mice were instilled with PBS (control) or 2.5 mg of crystalline silica. Lungs were collected at different time intervals after instillation. (A) OH-proline lung contents. ** *P *< 0.01 for comparison between control and silica-treated mice. (B) Cat transcripts were quantified by RT-real-time PCR on RNA extracted from lung tissue. Results were calculated as a ratio of Cat expression to β-actin expression and expressed as percentage of controls. Values of 5 mice in each group are presented as means ± SEM. All levels of Cat expression were significantly higher in silica-treated mice compared to control mice except for Cat L (all time points), and Cat B and S (at 3 days).

To identify lysosomal cysteine proteases potentially important in lung fibrosis, we first assessed the level of cathepsin expression during the development of silica-induced lung inflammation and fibrosis. C57BL/6 mice were instilled with 2.5 mg silica particles or PBS (control) and their lungs were collected after several time periods. We chose to concentrate on three time points representative of different stages of the silicotic disease in mice [29]. The early inflammatory reaction was monitored 3 days after instillation, the interface between the inflammatory and the fibrotic process after 1 month, and the established fibrosis at 2 months, as demonstrated later by the accumulation of collagen in the lung. The establishment of fibrosis in silica-treated mice was assessed by measuring lung OH-proline content, which reflects collagen deposition. We and others have shown the good correlation between this marker and histological fibrosis [30,31]. Two months after administration of silica particles, collagen significantly accumulated in silicotic lungs to levels twice that of healthy lungs (figure 1A). Cat K, L, S and B transcripts were quantified in the lungs by RT-real-time PCR at different time intervals after treatment. As shown in figure 1B, Cat K was the most highly upregulated cathepsin at all evaluated time points after silica instillation. Although Cat B and S were approximately overexpressed 2 fold after 1 and 2 months, Cat K reached levels up to 7 times higher than the controls. Cat K was found to be upregulated in mice lungs as already as 3 days after silica instillation. After its maximum was attained at the onset of the fibrogenic process, i.e. at the interface between inflammation and fibrosis (1 month), Cat K expression was maintained at a high level at the fibrotic stage (2 months). No change was detected for Cat L.

To assess whether Cat K transcript upregulation was associated with an increase of its enzymatic activity, we measured Cat K specific activity in whole lung homogenates. We concentrated on 2 months after instillation since Cat K expression is elevated at this time point and since 2 months represents the maximal collagen accumulation in lungs among time points studied. Lungs obtained 2 months after silica treatment of C57BL/6 mice showed significantly higher Cat K activity than lungs from control mice (respectively 0.144 ± 0.0087 fluorescence units and 0.0325 ± 0.0075 fluorescence units, *P *< 0.001, n ≥ 4).

### Cat K is specifically upregulated in response to fibrogenic particles

**Figure 2:**
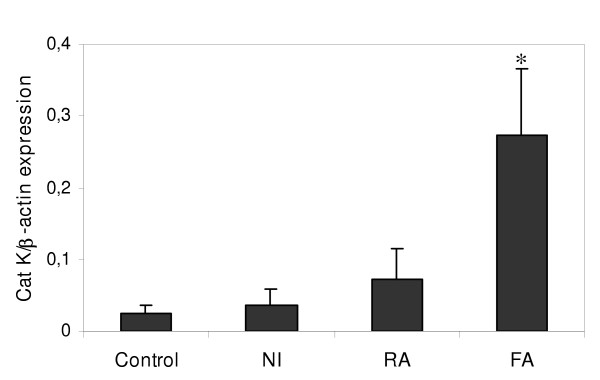
Pulmonary overexpression of Cat K is specific to a fibrotic response of the lung. Quantification of Cat K transcripts in lung tissue from NMRI mice instilled with PBS (control), WC (non-inflammatory model, NI), MnO_2 _(resolutive alveolitis model, RA) or silica (fibrosing alveolitis model, FA) 1 month after instillation. Values of 4 mice in each group are presented as means ± SEM. * *P *< 0.05 compared to control values.

In a comparative mouse model described previously [29], we tested whether the Cat K response was specific to the development of lung fibrosis. Therefore, NMRI mice were instilled with 2.5 mg of mineral particles inducing different lung responses. Tungsten carbide (WC) treatment is accompanied by no modification in inflammatory parameters and lung structure (noninflammatory model, NI). While silica induces a chronic alveolitis accompanied by a fibrogenic response (fibrosing alveolitis model, FA), manganese dioxide (MnO_2_) induces an acute lung inflammatory reaction without subsequent fibrosis (resolutive alveolitis model, RA). Interestingly, Cat K transcript levels were not significantly affected by the administration of inert (NI) or inflammatory particles (RA) whereas administration of silica strongly upregulated the pulmonary expression of Cat K 1 month after instillation (figure 2). Cat B and Cat S expressions were only slightly increased to similar levels both in the RA and FA models (data not shown).

### Cat K expression inversely correlates with the amplitude of the fibrotic response

**Figure 3:**
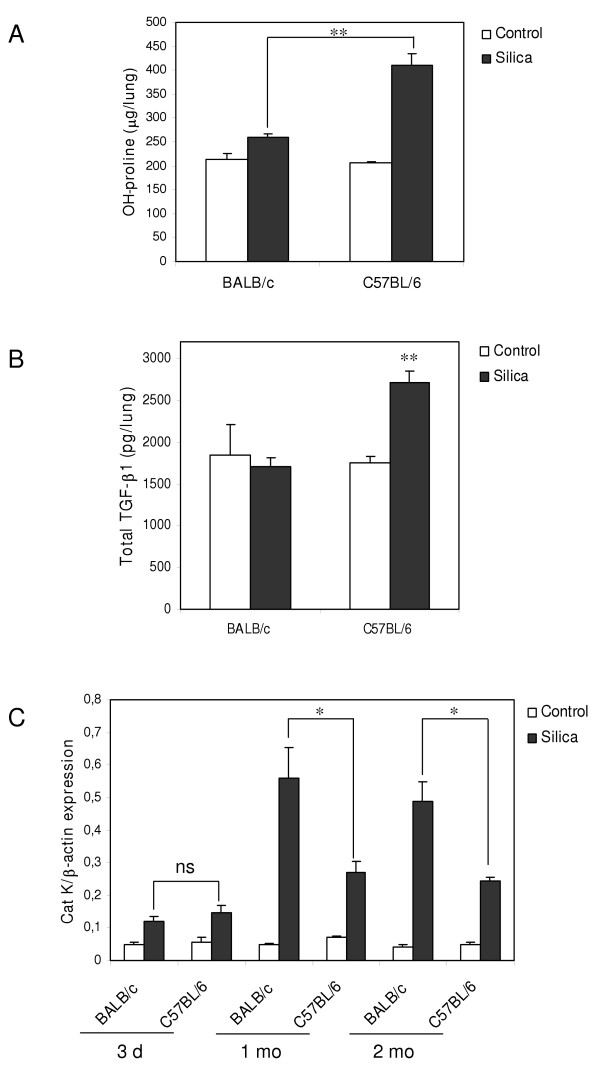
Cat K is more strongly upregulated in response to silica in "fibrosis-resistant" than in "fibrosis-prone" mice. BALB/c and C57BL/6 mice were instilled with PBS (control) or silica. (A) OH-proline lung contents 2 months after treatment. ** *P *< 0.01 for comparison of silica-treated mice between both strains. (B) Total TGF-β1 lung contents 1 month after instillation. ** *P *< 0.01 for comparison between silicotic and control lungs. (C) Cat K transcripts quantification on RNA extracted from lung tissue collected at different time intervals after instillation. Ns not significant, * *P *< 0.05 for comparison of silica-treated mice between strains at 1 and 2 months. Cat K expressions are not (significantly) different between all control conditions whereas it is significantly upregulated in all silica-treated groups compared to corresponding control groups. Values of 4 to 5 control mice and 5 to 6 silica-treated mice in each group are presented as means ± SEM.

We also examined Cat K expression in mice exhibiting low and high response to silica-induced pulmonary fibrosis. It is known that different strains of mice respond with variable degrees of susceptibilities to experimental factors inducing pulmonary fibrosis. Previous studies showed that, in response to silica instillation, the C57BL/6 strain displays a much more pronounced accumulation of collagen in the lung than the BALB/c strain [32]. To test the possible association between Cat K expression and the amplitude of fibrosis in these mouse strains, BALB/c and C57BL/6 mice were instilled with silica particles and analyzed as described above. OH-proline lung contents were quantified 2 months after treatment in order to verify the contrasting susceptibility of the both strains. Figure 3A shows that the accumulation of collagen in C57BL/6 fibrotic lungs was significantly more important after silica treatment than in BALB/c lungs. OH-proline levels in BALB/c nearly remained at the control level. TGF-β is thought to play a role in the differences of sensitivity in response to fibrosing agents because it was found to be more expressed in sensitive than in resistant mouse strains treated with bleomycin or irradiation [33-35]. We measured TGF-β lung content in response to silica to verify this hypothesis. One month after instillation, i.e. at the onset of the establishment of fibrosis, total TGF-β1 level was found to be significantly increased in C57BL/6 silicotic lungs compared to control lungs and TGF-β1 lung content in BALB/c mice remained unchanged (figure 3B). No significant differences were observed between control and treated mice 3 days and 2 months after instillation (data not shown). Interestingly, Cat K transcripts were differentially upregulated upon silica treatment in C57BL/6 and BALB/c lungs. Although Cat K mRNA levels at 1 and 2 months were increased in both strains in response to silica, the overexpression was significantly higher in resistant BALB/c mice than in sensitive C57BL/6 mice (figure 3C). No such difference between the both strains was observed after saline treatment (control situations) or 3 days after silica treatment. We concluded that pulmonary collagen contents and Cat K expression levels were inversely associated in this murine model of lung fibrosis.

### Pulmonary macrophages and fibroblasts overexpress Cat K in silicotic mice

**Figure 4:**
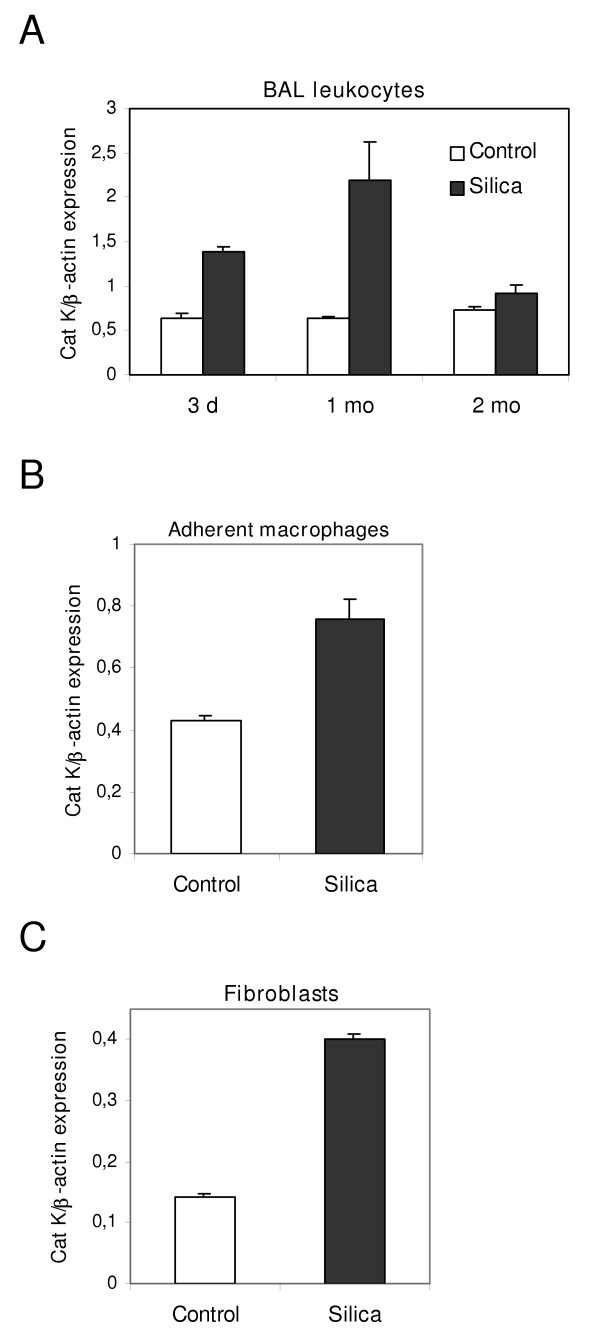
Cat K expression is higher in pulmonary macrophages and fibroblasts from silica-treated mice. Quantification of Cat K mRNA in pulmonary cells from C57BL/6 mice instilled with PBS (control) or silica. (A) Cat K expression in a pool of BAL leukocytes collected from 20 control mice and 10 silica-treated mice for each time point. (B) Cat K expression in adherent BAL cells (macrophages) collected 1 month after instillation of 40 control mice and 30 mice administered with silica. (C) Cat K expression in lung fibroblasts recovered 2 months after instillation from a pool of 9 control mice and 8 silica-treated mice. The results are representative of 3 independent experiments. (A), (B), (C) Values were measured 3 times for each condition and are presented as means ± SEM.

Pulmonary macrophages and fibroblasts play important roles in inflammatory and fibrogenic responses upon silica instillation by, respectively, producing fibrotic mediators and components of the organic matrix, such as collagen [36]. To determine which cells are responsible for the increase of Cat K mRNA in the lung, we studied the expression of this cathepsin in BAL leukocytes and fibroblasts from control and silica-instilled C57BL/6 mice. Cat K was found to be upregulated in BAL leukocytes collected 3 days and 1 month after instillation (Figure 4A). To further identify Cat K producing cells, lung macrophages were separated from other inflammatory cells by adherence. Figure 4B revealed that at 1 month adherent silicotic macrophages overexpress Cat K in comparison to adherent control macrophages, indicating that pulmonary macrophages are, at least in part, responsible for the Cat K upregulation. The fact that Cat K expression was markedly increased in the lung but not in BAL cells 2 months after silica instillation (figure 1B vs. figure 4A), suggested that other cells were involved in this process. In view of their major role in the production of extracellular organic matrices during fibrosis, Cat K expression was compared in isolated control and silicotic lung fibroblasts. Cat K mRNA levels were higher in fibroblasts from silica-treated mice at a fibrotic stage (2 months) than in control fibroblasts (figure 4C), demonstrating that lung fibroblasts are also able to overexpress Cat K in response to silica administration.

### TGF-β1 downregulates Cat K in mouse and human lung fibroblasts

**Figure 5:**
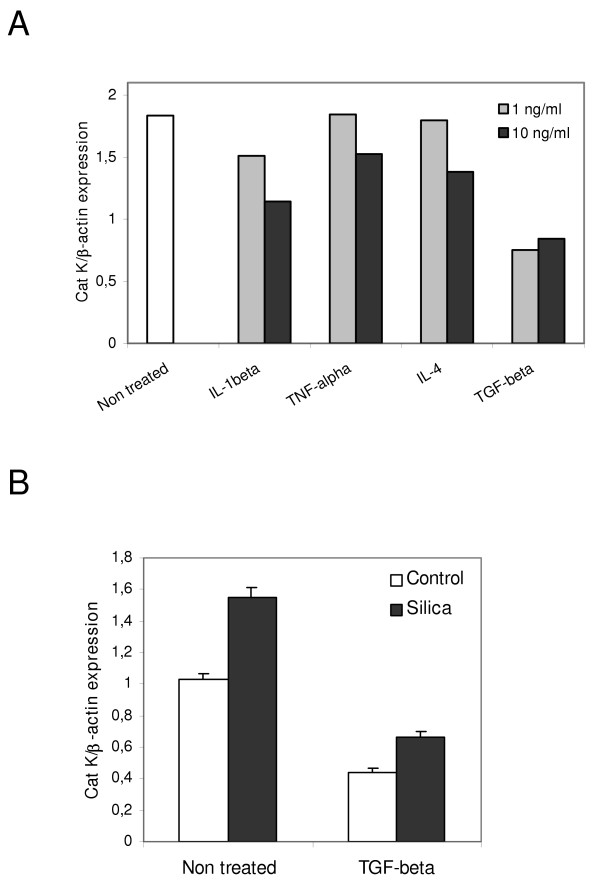
Cat K expression is reduced in response to TGF-β1 in control and silicotic mouse lung fibroblasts. Cat K mRNA quantification in pulmonary fibroblasts of C57BL/6 mice. (A) Control fibroblasts were incubated with 1 or 10 ng cytokine/ml. Bars represent the mean of triplicate measurements of Cat K expression on the same sample. The Cat K downregulation by TGF-β was reproduced in 4 independent experiments. (B) Fibroblasts from control (pool of 10 animals) and silicotic (pool of 7 animals, silica) mice collected 2 months after instillation and incubated at least in duplicates without (non-treated) or with 10 ng TGF-β1/ml (TGF-beta). The results are representative of 2 independent experiments (*P *< 0.001 in this experiment between non-treated and TGF-β treated fibroblasts, either control or silicotic). Values are presented as means ± SEM.

**Figure 6:**
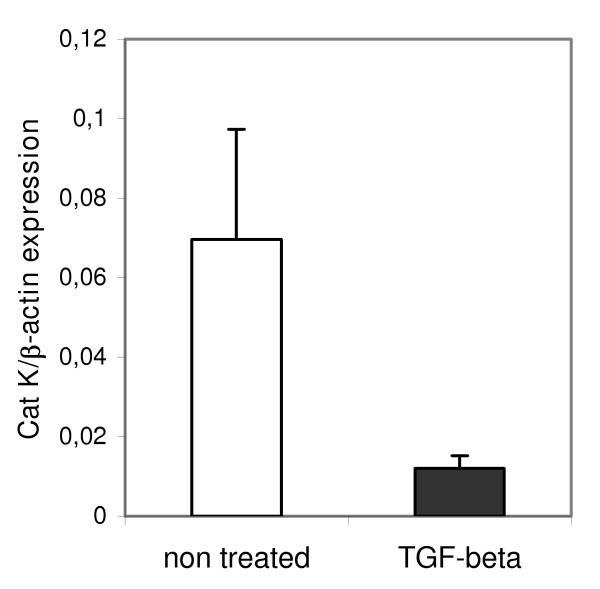
TGF-β1 downregulates Cat K mRNA expression in human lung fibroblasts. Quantification of Cat K mRNA in human pulmonary fibroblasts incubated with 10 ng TGF-β1/ml (TGF-beta) or non-treated. Values are presented as means ± SEM (5 culture-wells for each condition, *P *= 0.053).

Pulmonary macrophages and fibroblasts were identified as overproducing cells of Cat K transcripts. To identify mediators that could be responsible for the regulation of Cat K expression in the lung, cultured lung fibroblasts from healthy C57BL/6 mice were treated with several growth factors. While IL-1β, TNF-α and IL-4, known for their implication in the extension of lung fibrosis [37,38], had no or limited effect on Cat K expression, both concentrations of TGF-β1 (1 and 10 ng/ml) reduced Cat K expression (figure 5A). Moreover, TGF-β1 was also able to downregulate Cat K in lung fibroblasts purified from mice 2 months after silica instillation, i.e. at the fibrotic stage of the disease (figure 5B). No effect of this cytokine was observed on Cat K expression in pulmonary macrophages (data not shown). PGE2 and IL-9, two antifibrotic factors [25,39], did not affect Cat K expression in mouse fibroblasts (data not shown). We also measured the expression of Cat K in cultures of human lung fibroblasts treated or not with TGF-β1. Like in mice, a similar trend was observed in human lung fibroblasts obtained from a healthy individual where this growth factor reduced Cat K expression by 80% (*P *= 0.053, figure 6).

## Discussion

Lung fibrosis is characterized by tissue remodeling resulting from the imbalance between synthesis and degradation of extracellular organic matrices. While several mechanisms and mediators responsible for the stimulation or inhibition of matrix production have been widely studied, little information exists on the implication of proteases in the limitation of matrix accumulation in the fibrotic lung. In this study, we used a model of silica-induced lung fibrosis to screen the expression of four lysosomal cysteine proteases known for their collagenolytic activities in order to identify cathepsin(s) potentially important in the development of pulmonary fibrosis. Quantitative analysis of the cathepsin transcripts revealed Cat K as the most strongly upregulated protease in response to silica compared to Cat S, L and B. Several clues indicate that the overexpression of Cat K is intimately related to the fibrogenic process. First, the increased Cat K mRNA content in the lung of silica-treated mice was maximal after 1 month, i.e. when extracellular matrices start to accumulate, and remained elevated when fibrosis was clearly established (after 2 months). In the resolutive model of bleomycin-induced fibrosis, Cat K overexpression also slightly preceded collagen accumulation but returned to its basal level when the lung collagen content started to decrease (unpublished observation). These results show that Cat K expression is apparently modulated in parallel with collagen accumulation. Secondly, while silica particles induced a strong upregulation of Cat K in the lung, instillation of inert (WC) or inflammatory (MnO_2_) particles had no or little effect on its expression. These data, together with the fact that Cat K is also upregulated in patients suffering from different interstitial lung diseases and in mice instilled with bleomycin [23,24], support a particular role of Cat K in lung fibrotic diseases with various origins.

Two months after silica instillation, homogenates of silicotic lungs were shown to have a much higher Cat K activity than control lungs. This indicates that, despite the presence of endogenous cathepsin inhibitors in the cytoplasm of most cells [40], it is possible to measure changes in Cat K activity in this kind of sample. It also shows that pulmonary overexpression of Cat K transcripts correlates with an increase of its activity in lung homogenates 2 months after instillation, which corresponds to the maximal collagen accumulation.

We further characterized the contribution of Cat K in the development of lung fibrosis in the silica model by investigating its expression in "fibrosis-resistant" and "fibrosis-prone" mouse strains. We found higher levels of Cat K transcripts in the lungs of resistant (BALB/c) than sensitive (C57BL/6) mice in response to silica particles. These observations indicate that a high level of Cat K expression is associated with a low fibrotic response in the present model. Overall, our data, together with the fact that mice deficient for Cat K developed significantly more fibrosis than wild type counterparts after bleomycin instillation [24], indicate that Cat K might play a protective role in silica-induced lung fibrosis. This also illustrates that, during pulmonary fibrosis, not only profibrotic but also antifibrotic factors can be (over)produced and that fibrosis results from the inappropriate balance between these.

In bleomycin-induced lung fibrosis, qualitative immunostaining of lung sections have shown epithelial cells, macrophages and fibroblasts as Cat K producing cells while normal lungs expressed Cat K in epithelial cells and macrophages [24]. The same authors also showed that lung fibroblasts were the main contributors of Cat K overexpression in fibrotic human lungs. In silica-induced lung fibrosis, alveolar macrophages contribute to the installation of a chronic inflammation by producing several mediators leading to the recruitment and activation of other inflammatory cells [41-43]. Lung fibroblasts locate more downstream of the process by mainly overproducing components of the ECM, resulting in the excessive accumulation of ECM in the lung parenchyma [44]. Because of their central role in the induction of a fibrotic response induced by silica, Cat K expression was examined in these cell types. Both alveolar macrophages and lung fibroblasts were found to contribute to the overexpression of Cat K in silicotic lungs.

We confirm the overexpression of Cat K by fibrotic fibroblasts and suggest the macrophage as another overproducing cell in murine silicotic lungs. We can, however, not exclude that epithelial cells also contribute to the increased expression of Cat K in the lungs of these mice.

To identify regulators of Cat K expression, we tested the influence of several mediators involved in the pathogenesis of pulmonary fibrosis. We mainly concentrated our *in vitro *study on fibroblasts because this cell type has been found to overexpress Cat K in both human and mouse fibrotic lungs [24]. It is already well established that several factors, such as cytokines, can modify the expression or the secretion of cathepsins *in vitro *or *in vivo *[45-48]. We chose to test cytokines and factors known for their different activities on the development of lung fibrosis: proinflammatory (IL-1β and TNF-α), profibrotic (IL-4 and TGF-β) and antifibrotic mediators (IL-9 and PGE-2). None of the molecules tested *in vitro *could reproduce the overexpression of Cat K observed in the lungs of silica-treated mice. Some proteases are known to be upregulated by components of the organic extracellular matrix [49-51]. Fibronectin but not type I collagen has been found to increase Cat K mRNA expression in osteoclasts cultures [52]. Studies have shown that fibronectin starts to be overproduced earlier than collagen in the fibrotic process both in human fibrosis and mouse models of silica- and bleomycin-induced lung fibrosis [33,53,54]. It is therefore tempting to postulate that fibronectin or other components of the ECM could contribute to the upregulation of Cat K *in vivo*. The fact that two important proinflammatory cytokines did not modify Cat K expression and that inflammatory particles (MnO_2_) had only little effect on its expression compared to fibrogenic particles (silica), suggests that the inflammatory response induced by the instillation of silica probably plays a limited role in the induction of Cat K.

TGF-β is able to stimulate fibroblast proliferation and expression of ECM proteins by these cells [37]. It has also been shown to stimulate the production of the protease inhibitor TIMP-1 [55] and to downregulate some proteases, such as MMP-1, Cat B and L [45,55]. We show for the first time that the expression of a highly collagenolytic protease, Cat K, is repressed by this growth factor in collagen overproducing cells, i.e. fibrotic fibroblasts. The fact that TGF-β reduced Cat K expression in both control and silicotic mouse fibroblasts, as well as in human pulmonary fibroblasts suggests similar modes of regulation. Interestingly, although it has been shown that TGF-β represses Cat K expression in monocyte-derived osteoclasts [56], we did not find a similar effect on alveolar macrophages, although these cells seem to express both TGF-β receptors [57]. This might imply different TGF-β signaling pathways in both cell types.

TGF-β has been detected in several forms of lung fibrosis. In idiopathic pulmonary fibrosis, TGF-β located to activated foci [58]. Similarly, in silicosis, this growth factor was found to co-localize with silicotic granulomas both in rodents and humans [59,60]. We could therefore speculate that the presence of TGF-β at sites of high collagen production could repress the expression of Cat K by fibroblasts, limiting its potential antifibrotic activity. One argument in favor of this hypothesis is the stronger accumulation of Cat K transcripts in "fibrosis-resistant" BALB/c mice than in "fibrosis-prone" C57BL/6 mice in silica-instilled animals. Although the exact reasons for this difference in the fibrotic response between these two strains are still unclear, some evidence point to TGF-β as a key player in this phenomenon. First, because of its well-characterized profibrotic activity already mentioned. Secondly, we showed that TGF-β content increased in response to silica in the lungs of C57BL/6 but not BALB/c mice, confirming that TGF-β is more expressed in fibrosis-sensitive mouse strains than in fibrosis-resistant strains in response to fibrogenic stimuli [33-35]. Overall, these observations suggest that Cat K might be one of the downstream targets of TGF-β that could account for the difference of strain sensitivity, as it was recently proposed for TIMP-1 and the connective tissue growth factor [61,62].

## Conclusion

We have shown that the most potent collagenolytic mammalian protease, Cat K, is upregulated during a fibrotic process induced by the instillation of crystalline silica particles in mice. The expression level of Cat K was inversely associated with the susceptibility of murine strains to develop fibrosis in response to silica, suggesting that Cat K might contribute to limit lung fibrosis. Our *in vitro *and *in vivo *data support the view that the profibrotic growth factor TGF-β represses the expression of Cat K in lung fibroblasts to allow the development of fibrosis.

## Authors' contributions

SV carried out the experiments, participated in the experimental design and in the interpretation of data and wrote the manuscript. PM participated in the animal instillation and in some molecular biology experiments. FB performed the experiment on human fibroblasts. DL initiated the project, participated in the experimental design and in the interpretation of data and revised the manuscript critically. FH participated in the animal instillation, in the experimental design and in the interpretation of data and revised the manuscript critically.
